# Fine-Scale Genetic Structure in the United Arab Emirates Reflects Endogamous and Consanguineous Culture, Population History, and Geography

**DOI:** 10.1093/molbev/msac039

**Published:** 2022-02-22

**Authors:** Katherine S Elliott, Marc Haber, Hinda Daggag, George B Busby, Rizwan Sarwar, Derek Kennet, Michael Petraglia, Lawrence J Petherbridge, Parisa Yavari, Frauke U Heard-Bey, Bindu Shobi, Tariq Ghulam, Dalia Haj, Alia Al Tikriti, Alshafi Mohammad, Suma Antony, Maitha Alyileili, Shatha Alaydaroos, Evelyn Lau, Mark Butler, Arash Yavari, Julian C Knight, Houman Ashrafian, Maha T Barakat

**Affiliations:** 1 Wellcome Centre for Human Genetics, University of Oxford, Oxford, United Kingdom; 2 Institute of Cancer and Genomic Sciences, Centre for Computational Biology, University of Birmingham, Birmingham, United Kingdom; 3 Imperial College London Diabetes Centre, Abu Dhabi, UAE; 4 Big Data Institute, University of Oxford, Oxford, United Kingdom; 5 Experimental Therapeutics, Radcliffe Department of Medicine, University of Oxford, Oxford, United Kingdom; 6 Department of Archaeology, Durham University, Durham, United Kingdom; 7 Max Planck Institute for the Science of Human History, Jena, Germany; 8 National Archives, Abu Dhabi, UAE

**Keywords:** population genetics, ancestry, admixture, Middle East

## Abstract

The indigenous population of the United Arab Emirates (UAE) has a unique demographic and cultural history. Its tradition of endogamy and consanguinity is expected to produce genetic homogeneity and partitioning of gene pools while population movements and intercontinental trade are likely to have contributed to genetic diversity. Emiratis and neighboring populations of the Middle East have been underrepresented in the population genetics literature with few studies covering the broader genetic history of the Arabian Peninsula. Here, we genotyped 1,198 individuals from the seven Emirates using 1.7 million markers and by employing haplotype-based algorithms and admixture analyses, we reveal the fine-scale genetic structure of the Emirati population. Shared ancestry and gene flow with neighboring populations display their unique geographic position while increased intra- versus inter-Emirati kinship and sharing of uniparental haplogroups, reflect the endogamous and consanguineous cultural traditions of the Emirates and their tribes.

## Introduction

Situated at the crossroads of Africa, Europe, and Asia, and together with strong tribal social structure, the populations of the Arabian Peninsula provide an important ingredient to understanding human evolution and population admixture events. Studies unraveling the population history and genetic structure of Middle Eastern—and particularly Arabian Peninsula—populations have been relatively limited and Arabia is still underrepresented in such investigations, which may exacerbate health inequalities for diseases such as metabolic syndromes traits (Radwan et al. 2018), which are particularly prevalent in the Emirati population. Reports detail a complex melting pot of ancestries from the Levant and South Asia into the Arabian Peninsula, particularly in the East (Fernandes et al. 2019); African and Iranian content in Qataris (Hunter-Zinck et al. 2010); and Levantine contributions to Yemenites (Vyas et al. 2017). A recent study that generated physically phased whole-genome sequences for 137 individuals from the Levant and the Arabian Peninsula found significant genetic continuity in Arabia since Upper Paleolithic times including a Basal Eurasian ancestry derived from ancient Levantine hunter gatherers and Neolithic Iranians (Almarri et al. 2021).

Over the last 10,000 years, there is evidence of sequential climatic effects on Arabian populations. Aridification and long droughts led to regional abandonments and geographic relocation of groups (Preston et al. 2015; Petraglia et al. 2020), inducing bottlenecks in the Middle East observed in archaeology (Weiss et al. 1993; Magee 2014) and genetics (Almarri et al. 2021). Conversely, humid “greening” events facilitated population migrations and expansions (Petraglia et al. 2020). Movements promoted by sequential trade hubs, for example, pearl diving, or dependence on natural resources such as destruction of the Marib dam (Hill 1996) in Yemen, led to the migration of groups to Arabia, adopting nomadic lifestyles and consolidating kinships structures (Heard-Bey 2004). However, the impact of all these events on the genetic diversity of local populations is not well understood yet.

In recent history, the discovery of oil in 1959 transformed the population of UAE, growing from 87,000 to 9.9 million (November 2020), largely due to the influx of expatriates primarily from the Indian subcontinent. Emiratis account for an estimated 11% of the current population. Against this complex flux that would be expected to promote genetic admixture, social and, cultural factors are proposed to have restricted gene flow and diversity (Zayed 2016). There is a high prevalence of consanguineous marriage estimated to be as high as 20–50% of all marriages in the Middle East, particularly when first male marriages are considered (Tadmouri et al. 2009; Hamamy 2012). In many countries, multiethnic immigration has increased mixing of disparate gene pools. However, the Emirati population observes tribal endogamy and marriage with non-Emiratis is discouraged. These practices provide the opportunity for the preservation of fine-scale genetic structure whose characterization can yield insights into the early founding gene pools and population history and are relevant to future medical studies.

## Results

### Data Set, Genotyping, and Homozygosity

**Fig. 1.**
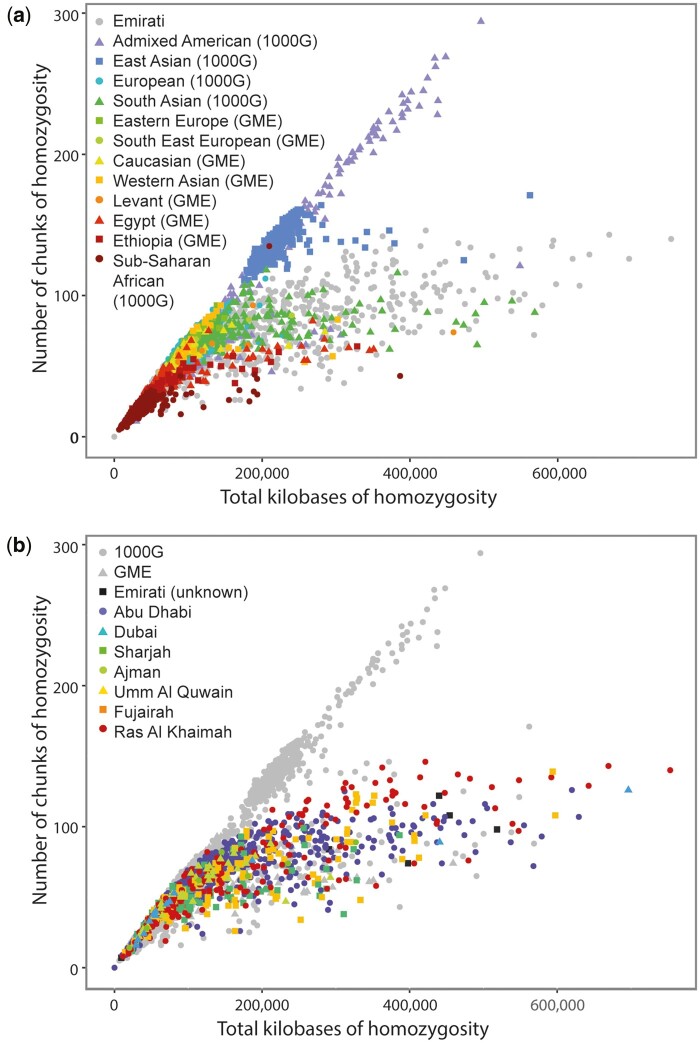
Homozygosity of Emiratis compared with world populations. (*a*, *b*) Plots show number of total kilobases of homozygous DNA (x-axis) against number of homozygous segments (y-axis). A small number of segments and little homozygosity are characteristic of African populations. A large amount of homozygosity broken into many small segments is typical of founder populations and can be observed in some Admixed Americans. Long tracts of homozygosity are indicative of the consanguineous cultures found in Middle Eastern populations. Data are also shown for participating individuals by Emirate of origin.

Here we report the fine-scale genetic structure and ancestry of the Emirati population based on an analysis of 1,198 individuals from the UAE, a federation of seven Emirates (Abu Dhabi, Dubai, Sharjah, Ajman, Umm Al Quwain, Fujairah, and Ras Al Khaimah) (supplementary table S1, Supplementary Material online). Dubai and Ajman were excluded from some analyses due to small sample size. For each individual, a total of 1,742,591 variants were genotyped on the Multi-Ethnic Global-8 v1 chip (Illumina). We found that Emiratis had a high proportion of individuals with longer tracts of homozygous segments (fig. 1*a* and *b* and supplementary fig. S1, Supplementary Material online) consistent with a high degree of consanguinity. Emirati populations also showed a high degree of kinship compared with other populations (supplementary fig. S2, Supplementary Material online), and there is considerably greater intra-Emirati kinship compared with inter-Emirati kinship, reflecting the endogamous, tribal culture of the Emirati populations (Heard-Bey 2004) (supplementary fig. S3, Supplementary Material online).

### Population Structure and Admixture

**Fig. 2.**
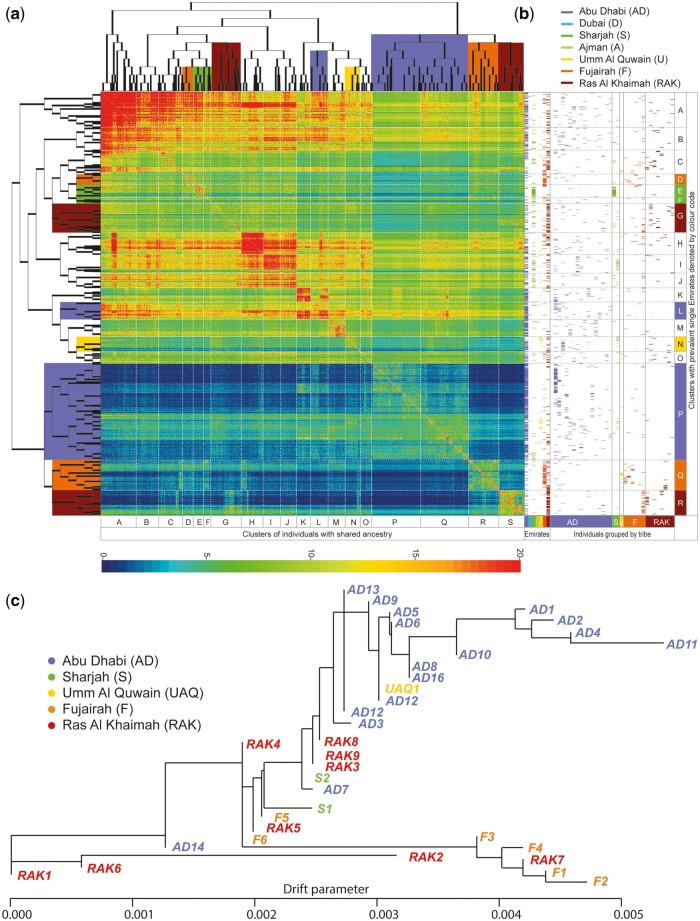
Shared ancestry between Emirati individuals. (*a*) fineSTRUCTURE was used to quantify pairwise contribution of haplotype chunks between each Emirati individual. The heatmap shows the number of chunks shared between each pair of individuals with red depicting greater sharing and blue less sharing. Each line (horizontally and vertically) represents one of 1,198 Emiratis. All Emiratis were clustered into 107 relationship groups which are nodes on the tree calculated to illustrate the ancestral relationship between each of the clusters. (*b*) Each column on the left shows individuals in each Emirate and tribe to illustrate clustering of Emirati populations and families with some branches of the tree. Branches of the tree are color coded and labeled with letters to allow description of branches showing clustering of one Emirate or tribe. (*c*) Treemix analysis inferring Emirati tribe branch points shows different tribes within the same Emirate cluster together under common branch points. This cosegregation is particularly striking for Abu Dhabi Emirati populations at the top of the tree and Fujairah Emirati populations at the bottom.

We were interested in elucidating the fine-scale genetic structure within the Emirate and thus we performed a ChromoPainter (Lawson et al. 2012) analysis where we reconstructed each Emirati individual’s haplotypes as a mosaic of a set of donor individuals that only included other Emiratis. We used the resultant pairwise copying matrix with fineSTRUCTURE to group individuals into clusters on the basis of their shared haplotypes (fig. 2*a* and *b*). The values in this matrix represent the amount of haplotype sharing between individuals and show that in many instances clustering brought together individuals from the same Emirate. We annotated the main branches with letters A to R to compare them and we found that many clusters were dominated by individuals from single Emirati populations; for example, branches D and Q by Fujairah (*P* = 3.97 × 10^−5^ and 1.4 × 10^−29^_,_ respectively) (Mann–Whitney *U* test), E and R by Ras Al Khaimah (*P* = 6.33 × 10^−9^ and 1.59 × 10^−24^, respectively), I and N by Umm Al Quwain (*P* = 1.45 × 10^−24^ and 0.0042, respectively), M by Dubai (*P* = 2.85 × 10^−8^), and P by Abu Dhabi (*P* = 1.85 × 10^−32^). These results are additionally supported by a population relationship tree inferred using Treemix (Pickrell and Pritchard 2012) showing that the different Emirati tribes mostly cluster within the same Emirate branch (fig. 2*c*).

We next investigated the genetic relationship of the Emiratis to worldwide populations by combining our samples with the 1000G data and with published data from Sub-Saharan Africa, the Middle East, Europe, the Caucasus, and South Asia (Behar et al. 2010; Pagani et al. 2015; Sudmant et al. 2015; The 1000 Genomes Project Consortium et al. 2015; Yunusbayev et al. 2015; Pagani et al. 2016) (supplementary tables S1–S5, Supplementary Material online). This allowed us to understand the current genetic landscape in the Emirates and how it is related to neighboring populations. In this work, we refer to populations that might have been genetically relevant to the Emirates but not part of the 1000G data as the Greater Middle East (GME) super-populations (see supplementary table S4, Supplementary Material online)—a term used here strictly to differentiate the data sets rather than describe political or historical borders.

**Fig. 3.**
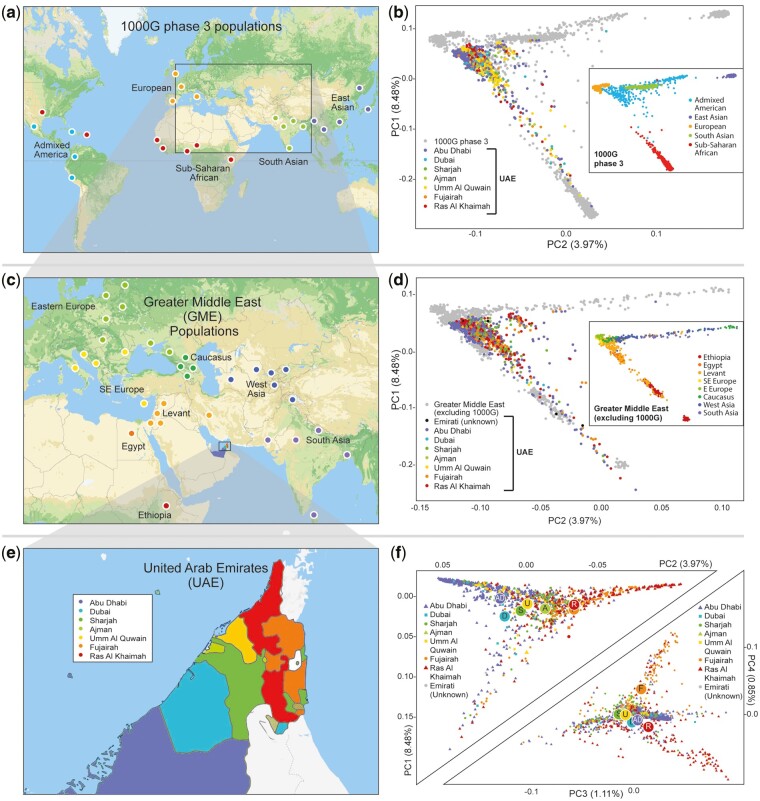
PCA of 1,198 Emiratis and neighboring populations. (*a*) Map showing geographic distribution of the 1000G phase 3 populations. Note the gap in samples from the Middle East. (*b*) PCA showing the Emirati individuals in our study cohort projected onto 1000G variation and occupying a region close to Europeans but drawn toward Africans and South Asians, presumably reflecting admixture with these populations. (*c*) Geographic distribution of GME populations and (*d*) PCA showing the Emirati are close to the Caucasus, West Asia, and SE Europe populations. (*e*) Map of the United Arab Emirati populations showing the borders of the seven individual Emirati populations (for illustrative purposes, the Southern region of the largest Emirate, Abu Dhabi, is not shown). (*f*) PCA restricted to the Emirati individuals and grouped by Emirate of origin shows a strong correlation with East/West geographical distribution of the Emirati populations along PC2. Large colored dots show average values for each Emirate. Tapering of distribution along PC1 is consistent with an increasing degree of African ancestry. Variance explained by each PC is shown in brackets. The x and y axes have different scales.

Principal components analysis (PCA) (Price et al. 2006) showed that the relationship of Emirati individuals to 1000G populations reflects the Emirates’ intercontinental location between Africa, Europe, and Asia (fig. 3*b*). Emiratis form a distinct cluster, located proximal to the European populations on PCs 1 and 2 but drawn toward Sub-Saharan African populations while a subset of the Emiratis appears drawn toward South Asians. We also note here that some individuals completely overlapped with Africans and Central Asians reflecting their recent origin from these populations.

When we examined the pattern of distribution of individual Emirates within the 1000G and GME joint PCA analyses, we observed inter-Emirati genetic structure (fig. 3*e* and *f*). This is markedly demonstrated by PCA restricted to Emiratis, which displays a distinct pattern that loosely correlated with geography, from Abu Dhabi (Western UAE) through Dubai, Sharjah, Ajman, and Umm Al Quwain Emirates in the center to the Easternmost Emirates of Fujairah and Ras Al Khaimah (fig. 3*f*).

Population tree inferred using Treemix (Pickrell and Pritchard 2012) showed that all Emirates, together with Qatar, cluster on a branch and receive African gene flow, probably from diverse sources as it was previously suggested (Hellenthal et al. 2014; Almarri et al. 2021) (supplementary fig. S4, Supplementary Material online). We estimate using admixture-induced linkage disequilibrium (ALDER) (Loh et al. 2013) that the African ancestry was already present in the Emirates around 1,000 years ago (supplementary fig. S5*a*, Supplementary Material online) but its influx appears to have been a continuous process until very recent times (supplementary fig. S5*b*, Supplementary Material online) consistent with our findings from the PCA. Similarly, Central Asian admixture can be detected starting 2,900 years ago (supplementary fig. S5*c*, Supplementary Material online) and continued until more recent times (supplementary fig. S5*d*, Supplementary Material online).

**Fig. 4.**
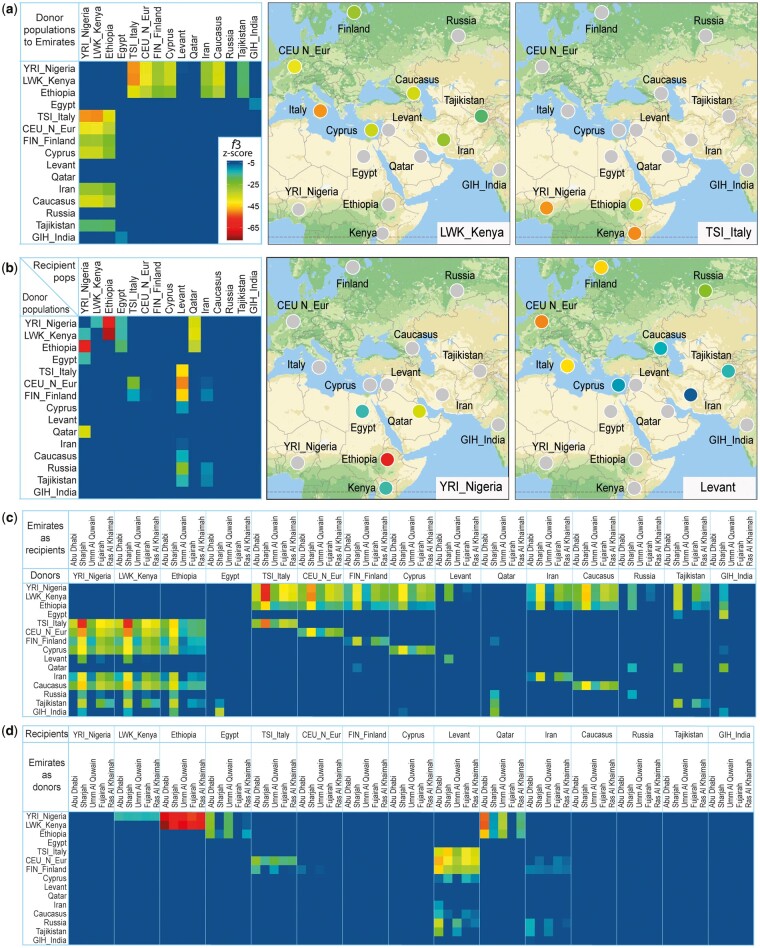
f3 admixture analyses of Emirati populations and GME populations. Heatmap panels show admixture contributions from donor pairs to recipient populations. Colored circles on the map represent f3-statistics with *Z*-scores < −5 (significant admixture) while gray circles represent tests with *Z*-scores > −5. (*a*) Combined Emirati populations as recipients of admixture from GME populations shows the lowest *Z*-scores are for African and European populations as pairs of ancestry sources, particularly Southern Europe and East Africa. Map show admixture in combination with LWK_Kenya and TSI_Italy. (*b*) Combined Emirati populations as source of admixture reveals significant admixture in Ethiopia, and to a lesser degree into the Levant and Qatar. Maps show Emirati admixture combined with other populations into YRI_Nigeria and the Levant. (*c*) Heatmap showing the five largest Emirate sample sets as targets of pairwise admixture. Sharjah Emiratis appear in this test more admixed than other Emirati populations. (*d*) Heatmap showing the five largest Emirate sample sets as sources of pairwise admixture. Compared with other Emirati populations, Abu Dhabi represent a significant source of ancestry particularly to Ethiopia and Qatar. Color coding for heatmaps and map colored dots is for *f3* statistics *Z*-scores < −5 as shown of the heatmap of panel *a*. It is important to note here that the tested populations should be considered as proxies to the ancestral populations involved in the admixture events and therefore need not to be themselves the sources of ancestries or gene flow.

We next tested admixture using f3-statistics (Reich et al. 2009) placing the Emiratis as either target or source of admixture in combination with GME populations (fig. 4). We initially performed an analysis with 49 populations which we then reduced to a smaller set of 15 populations to represent the most significant contributing regions. The Emiratis appear in these tests as significant source of ancestry to many Eurasian and East African populations suggesting an autochthonous component related to ancient Middle Easterners is retained in the genetic landscape. As targets of admixture (fig. 4*a*), the Emirati populations have significant African ancestry as shown in our PCA, Treemix (supplementary fig. S4, Supplementary Material online), and ALDER results (supplementary fig. S5, Supplementary Material online).

Next, the five largest Emirate groups were analyzed separately, which allowed observation of distinct ancestral differences between them. The Sharjah Emirate appears in this test to have more significant African/Eurasian admixture compared with the other Emirates (fig. 4*c*). When tested as sources for admixture, the five largest Emirates also showed some differences (fig. 4*d*). For example, Abu Dhabi was the top significant source of ancestry shared with Ethiopia, Qatar, and the Levant. We should note here that the Emiratis in these tests are representing the ancient Middle Eastern populations and their movements. One example is the Eurasian admixture into Ethiopia, which we estimate using the Emiratis and Yoruba as references (the Amhara population as a target) to have occurred around 82 ± 4 generations ago (Z-score = 14.62) similar to dates found by Pagani et al. (2012).

### Y Chromosome and Mitochondrial Haplogroup Analysis

We next inferred Y chromosome (Poznik 2016) and mitochondrial (Weissensteiner et al. 2016) haplogroups in individuals in the pihat < 0.5 cohort (supplementary table S6, Supplementary Material online) and observed differences in the frequency of haplogroups between the Emirates. Rather than being driven by single tribes that may have greater kinship, these differences were seen across multiple tribes, further illustrating the endogamous nature of the Emirati culture. The largest group of Y chromosomes belonged to J1 haplogroups (P58–17% and L65.2 15%), which is frequent in the Arabian Peninsula reaching 73% in Yemen (Abu-Amero et al. 2009) and also distributed along the Fertile Crescent (Chiaroni et al. 2010; Dogan et al. 2017), and the E1b1-P2 haplogroup (22%), where the highest contemporary frequencies are in East Africa and the Horn of Africa (Cruciani et al. 2007; Trombetta et al. 2011). Additionally, we found in the Emirates the African A and B haplogroups and the predominantly South Asian haplogroups L1 and R2, mirroring admixture patterns we detected from genome-wide analysis. Similarly, we found African mitochondrial (mtDNA) haplogroup L in the Emirate with a frequency of ∼15%, almost twice as high as previously reported from the Emirates (Aljasmi et al. 2020). Although the South Asian haplogroups U2–U4 were ∼8% in our data set. These results confirm previous findings that the region has been a significant receptor of human migrations (Abu-Amero et al. 2009; Černý et al. 2016).

We observed clear patrilineal and matrilineal differences between the Emirates (supplementary fig. S5, Supplementary Material online). More specifically, the majority of the Y chromosomes from Fujairah individuals belonged to haplogroup R1b1a2a-L23 (45%). This clade is not frequent in the Middle East but was predominant across multiple Fujairah tribes in our sample. Conversely, the J1a2b2a1 subclade of the Middle Eastern J1a2b-P58 haplogroup seen in other Emirates (18%), was completely absent in Fujairah Emiratis. In our cohort, two Ras Al Khaimah tribes have a predominance of the E1b1b1b2a-M123 Y haplogroup, which is found in 5–8% of males in regional populations (Cruciani et al. 2007). In contrast, volunteers originating from Abu Dhabi had significant contribution from some rare haplogroups such as the T1a1a1a1a-P77 subclade seen in 17% of males, in particular one tribe, in which 21/38 males (55%) carried the haplotype although the regional frequency of this haplogroup is 5–8% (Abu-Amero et al. 2009). This tribe is part of the Bani Yas tribe who migrated east from Central Arabia after discovering sweet water in the Liwa region in the south of Abu Dhabi where they settled at numerous oases retaining their tribal coherence (Heard-Bey 2004).

We observe considerable contrast in the distribution of Y and mitochondrial haplogroup frequencies within families originating from the same Emirate (supplementary fig. S6, Supplementary Material online). Some of the haplogroups that are present in several families of the same Emirate, especially the Fujairah volunteers, were in many instances either absent or at very low frequency in other Emirati populations, probably illustrative of consanguinity and endogamy.

## Discussion

We have investigated the genome-wide diversity of the Emirati population and found that both ancient and more recent demographic events have contributed to the genetic formation and structure of the population. Our admixture tests suggest that the population retains an autochthonous Middle Eastern ancestry supplemented with African and South Asian ancestries. The Emirati population captures admixture events that have occurred thousands of years ago, possibly related to movement of people in the Middle East after major cultural transitions such as the invention of agriculture or more recent movements related to climate change and desertification of the region in the past 6,000 years ago (Petraglia et al. 2020; Almarri et al. 2021). However, we found in our data set individuals who were genetically identical to present-day Africans or Central/South Asians, suggesting gene flow into the Emirates is still an ongoing process. Yet, the spread of these ancestries across the tribes of the Emirate appears to have been restrained by a tradition of endogamy and consanguinity. We show that the specific tribal culture in the Emirates has created genetic structure in the population and that inter-Emirati genetic differences broadly reflect the geographical locations of the individual Emirati populations (fig. 4*c*) but in addition, family and tribe affiliation within the Emirate also contributed to genetic structure. The influence of the endogamous culture within the separate Emirates is probably best illustrated by the spread of specific uniparental lineages within some tribes but not others in the same Emirates—a pattern also seen elsewhere in endogamous tribes of the Middle East such as in Yemen (Raaum et al. 2013). Consanguinity has also resulted in high levels of homozygosity in individuals’ genomes characterized by long ROH segments, which could have consequence on health and disease (Ceballos, Joshi, et al. 2018). On the other hand, consanguinity has probably enriched rare functional variants in this population and thus it presents an opportunity to study the genetic architecture of complex human traits in the future (Xue et al. 2017).

Our study represents the first fine-scale genetic analysis of the Emirati population building on broader population structures revealed by other studies of the Arabian Peninsula. We uncover a unique and distinct genomic architecture and provide new insights into the ancestry of these populations and the social dynamics influencing their diversity. Complex disease risk alleles, rare and undetectable in other populations, may be amplified by cultural factors to discoverable levels, providing novel insights into disease mechanisms applicable to all populations. For Emirati populations, this will have important implications for future efforts to understand genetic risk and facilitating development of population-specific therapeutic interventions (Zayed 2016) including for current health challenges such as metabolic syndromes traits (Radwan et al. 2018) as well as improving understanding of drug responses. Such efforts reduce the potential for bias in representation of Emirati and other understudied populations worldwide that can exacerbate disease and generate healthcare disparities (Wojcik et al. 2019), and promote genomics-driven precision medicine approaches through a deeper understanding of population-specific genetic variation.

## Materials and Methods

### Sample Collection and Selection

Samples from Emirati individuals were collected through the Imperial College London Diabetes Centre (ICLDC), Abu Dhabi, UAE. The cohort reflects volunteers attending ICLDC for periodic health check-ups regardless of any disease status. Emirate of origin of each volunteer was assigned as per their Emirati Family Book (a UAE Government-issued legal document) or was reported by the individual. Tribes were defined by family name. All volunteers gave written informed consent for inclusion in the study. Ethical approval was given by the ICLDC Research Ethics Committee (IREC 011).

Abu Dhabi, Sharjah, Umm Al Quwain, Fujairah, and Ras al Khaimah assigned volunteers had the largest sample sizes and it is for these Emirati populations that we discuss the most significant results in the main text of the manuscript. The two remaining Emirati populations (Dubai and Ajman) for whom we had fewer volunteers (supplementary table S1, Supplementary Material online) were omitted from some analyses. Genomic DNA was extracted from whole blood using Qiagen’s PAXgene blood DNA isolation kits.

### Genotyping and QC

Samples were genotyped using Illumina’s Multi-Ethnic Global-8 v1 chip (Illumina). Genotyping data were processed and quality controlled using PLINK (Purcell et al. 2007) standard methods outlined in Anderson et al. (2010) as follows (Anderson et al. 2010). Samples were checked for consistency of reported sex with genotypic sex using plink –check-sex. Of the errors reported, samples were either reassigned to the opposite sex, reassigned to the ambiguous sex code (0), or remained assigned to their reported sex. Most samples failing were female and because plink –check-sex works by detecting excess homozygosity in males due to hemizygosity of the X and Y genotypes, it was postulated that the high level of consanguinity was leading to a higher proportion of homozygous genotypes and consequently resulting in sex check failure for some females. Pihat was generated using the “–genome” function of plink to generate a measure of identity by descent (IBD) pairwise between individuals in each group. The minimum number of individuals possible were removed to produce a cohort with no pairwise pihat values >0.5, to reduce relatedness. We acknowledge this greater than the pihat 0.2 threshold used in outbred populations, but this would not be appropriate for a population with a high degree of endogamy and consanguinity such as the Emirates. Individuals or SNPs with greater than 5% missing genotypes were excluded. PCA was used to identify extreme sample outliers which representing genotyping errors.

### Homozygosity

When measuring homozygosity, we used the plink –homoz together with the –homozyg-window-het flag to allow 0, 1, 2, or 3 heterozygous calls to be present in homozygous windows which was shown to be useful when comparing runs of homozygosity size and sum between data sets using different technologies (Ceballos, Hazelhurst, et al. 2018) (fig. 1 and supplementary fig. S1, Supplementary Material online and legends).

### Principal Components Analysis: Projection onto 1000G Weightings

When performing PCA analysis weighting principal components across all combined data sets, the high number of Emirati individuals distorts the distribution. To address this, we projected non-1000G populations (supplementary table S4, Supplementary Material online) onto 1000G weightings calculated from 1000G populations which represent relatively even numbers of individuals for each population globally (supplementary table S3, Supplementary Material online). Emiratis were projected onto 1000G weightings as follows:

Export allele frequencies and PCA variant weights from 1000G reference data set.

plink2 –bfile hapmap –freq –pca var-wts –out pca_hapmap

Use –score to compute the necessary dot products with the variant weights.

plink2 –bfile Emiratis –read-freq pca_hapmap.afreq –score pca_hapmap.eigenvec.var 2 3 header-read no-mean-imputation variance-normalize –score-col-nums 5-14 –out pca_proj_mydata

from https://groups.google.com/forum/#!topic/plink2-users/W6DL5-hs_Q4

### Phasing

We used SHAPEITv2 (Delaneau and Zagury 2012) to generate phased chromosomes for each individual. SHAPEITv2 conditions the underlying hidden Markov model (HMM) (Li and Stephens 2003) to estimate haplotypes from genotype data. We split our data set by chromosome and phased all individuals simultaneously, and used the most likely pairs of haplotypes (using the –output-max option) for each individual for downstream applications. We performed 30 iterations of the MCMC and used default values for all other parameters.

### Painting Chromosomes with CHROMOPAINTER

We used fineSTRUCTURE (Lawson et al. 2012) to identify fine-scale population structure and to identify high level relationships between the Emirati populations. The initial step of a fineSTRUCTURE analysis involves “painting” phased chromosomes sequentially using an updated implementation of a model initially introduced by Li and Stephens (2003) and which is exploited by the CHROMOPAINTER package (Lawson et al. 2012). The Li and Stephens copying model explicitly relates linkage disequilibrium to the underlying recombination process and CHROMOPAINTER uses an approximate method to reconstruct each “recipient” individual’s genome as a series of recombination “chunks” from a set of sample “donor” individuals. The aim of this approach is to identify, at each SNP as we move along the genome, the closest relative genome among the members of the donor sample. Because of recombination, the identity of the closest relative will change depending on the admixture history between individual genomes. Even distantly related populations share some genetic ancestry because most human genetic variation is shared (International HapMap 3 Consortium et al. 2010; Ralph and Coop 2013), but the amount of shared ancestry can differ widely. We use the term “painting” here to refer to the application of a different label to each of the donors, such that—conceptually—each donor is represented by a different color. Donors may be colored individually, or in groups based on a priori defined labels, such as the geographic population that they come from. By recovering the changing identity of the closest ancestor along chromosomes, we can understand the varying contributions of different donor groups to a given population, and by understanding the distribution of these chunks we can begin to uncover the historical relationships between groups.

Copying vector summaries generated from painted chromosomes describe how populations relate to one another in terms of the relative time to a common shared ancestor, subsequent recent admixture, and population-specific drift (Hellenthal et al. 2014; Leslie et al. 2015). Given a number of potential admixing donor populations, a key step in assessing the extent of admixture in a given individual or population is to identify which of these donors is relevant to the ancestral history of the populations. Following Busby et al. (2016), we used the nnls package in R to perform a non-negative least squares regression on the Emirati individual and population copying vectors, using copying vectors from the 24 non-Emirati donor populations as predictors. The coefficients of this regression represent ancestry proportions for a given recipient individual (or population). These mixing coefficients describe a recipient individual or population’s DNA as a linear combination of the set of donor populations.

### Using Painted Chromosomes to Infer Shared Ancestry

We performed an initial chromosome painting analysis where we painted each Emirati individual as a recipient using all individuals in the “GLOBAL” data set as donors. To generate figure 5*a* and *b*, we show the average length of shared chromosomes with each global region averaged across all individuals in an Emirate.

### Using Painted Chromosomes with fineSTRUCTURE

FineSTRUCTURE groups individuals on the basis of shared copying vectors. We performed a second painting analysis using only the Emirati individuals and painted each individual as a recipient with every other as a donor.

We ran fineSTRUCTURE for 100,000 iterations with a burn in of 10,000,000 and sampling every 10,000 iteration. We chose the maximum a posteriori run of these 100,000 iterations as our final clustering results and reordered the coancestry matrix with this clustering. We ran fineSTRUCTURE’s tree building algorithm for 100,000 iterations and used the output tree in figure 2.

### Admixture Tests

Tests of admixture were performed (*f3-statistics*) (Reich et al. 2009) placing Emiratis as either recipients or sources of admixture in combination with GME populations (Patterson et al. 2012; Skoglund et al. 2015) (fig. 4). We analyzed 49 populations with the five Emirates for which we had the largest samples and then reduced to a smaller set of 15 populations which were the most significant representatives of regions that showed evidence of admixture with the Emirates. All tests of admixture were performed with the Popstats software (Skoglund et al. 2015) (https://github.com/pontussk/popstats, last accessed February 17, 2022).

### Inference of Population Maximum-Likelihood Tree and Migration Events

Treemix (Pickrell and Pritchard 2012) (https://bitbucket.org/nygcresearch/treemix/wiki/Home, last accessed February 17, 2022) was used to infer a maximum-likelihood tree with migration events for the five largest Emirate samples and GME populations. The same analysis was performed for the Emirati tribes.

### Estimating Admixture Dates

ALDER (Loh et al. 2013) was used to date admixture time with parameters mindis: 0.005, binsize: 0.0005, and a generation time of 29 years. CEU, YRI, and ITU were set as references and 100 Emiratis were randomly selected from the data set to test admixture. Another 100 Emiratis were selected after stringent outlier removal using smartpca (Patterson et al. 2006) with outliersigmathresh: 3 and also tested for admixture time.

### Y Haplogroup Assignment for Patrilineal Inheritance

Patrilineal inheritance was determined from the Y-chromosome genotypes of 568 Emirati males using YHaplo (Poznik 2016) (https://github.com/23andMe/yhaplo, last accessed February 17, 2022).

### Mitochondrial Haplogroup Assignment for Matrilineal Inheritance

Matrilineal inheritance was determined from the mitochondrial genotypes of 630 Emirati females using HaploGrep 2 (Weissensteiner et al. 2016) (https://haplogrep.uibk.ac.at/, last accessed February 17, 2022).

### Y Chromosome and Mitochondrial Variants Present in Our Data Set

Mitochondrial and Y chromosome variants present in our data are listed in supplementary excel file Y_chr_and_mito_ genotypes_Illumina_ Multi_Ethnic_Global_Array_variants.xlsx, Supplementary Material online. “0” alleles represent alleles not seen in our data set.

### Statistical Analysis

Statistical analysis and plotting was performed using R, Rstudio, Python, and Microsoft excel.

### Publicly Available Data Sets and Resources

1000G, http://www.internationalgenome.org (last accessed February 17, 2022)
Ethiopian and Egyptian genomes, http://www.sciencedirect.com/science/article/pii/S0002929715001561 (last accessed February 17, 2022)
Whole genome diversity panels, https://www.simonsfoundation.org/ (last accessed February 17, 2022)
Estonian Biocentre, http://evolbio.ut.ee/ (last accessed February 17, 2022)
Wikipedia, https://www.wikipedia.org/ (last accessed February 17, 2022)

### Tools and Software

Python, https://www.python.org/ (last accessed February 17, 2022)
R, https://www.r-project.org/ (last accessed February 17, 2022)
Rstudio, https://www.rstudio.com/ (last accessed February 17, 2022)
PLINK, http://www.cog-genomics.org/plink2/ (last accessed February 17, 2022)
fineSTRUCTURE and ChromoPainter, https://people.maths.bris.ac.uk/~madjl/finestructure/index.html (last accessed February 17, 2022)
treemix: https://bitbucket.org/nygcresearch/treemix/wiki/Home (last accessed February 17, 2022)
Yhaplo, https://github.com/23andMe/yhaplo (last accessed February 17, 2022)
HaploGrep2, http://haplogrep.uibk.ac.at/ (last accessed February 17, 2022)
FigTree (Dendrogram in fig. 4), http://tree.bio.ed.ac.uk/software/figtree (last accessed February 17, 2022);
Maps in figures 1, 2, and 6, https://snazzymaps.com/ (last accessed February 17, 2022)

### Managed Data Access

Access can be granted by contacting the data access committee: Katherine Elliott (corresponding author; kelliott@well.ox.ac.uk), Hinda Daggag, Alia Al Tikriti, Houman Ashrafian, and Maha Barakat (chair). Access will be subject to the rules and regulations of the Emirate of Abu Dhabi, at the time of request. Access-required membership and/or appropriate use agreement with Imperial College London Diabetes Centre (ICLDC) will also apply.

## Supplementary Material

Supplementary data are available at *Molecular Biology and Evolution* online.

## Supplementary Material

msac039_Supplementary_Data
