# Supplementary material for: Fine-Scale Genetic Structure in the United Arab Emirates Reflects Endogamous and Consanguineous Culture, Population History, and Geography
Source: Mol Biol Evol. 2022 Feb 22;39(3):msac039. doi: 10.1093/molbev/msac039 (PMC8911814; doi:10.1093/molbev/msac039)
Supplement: msac039_Supplementary_Data [file msac039_supplementary_data.zip › Elliott_Emirati_pop_gen_supp_final.docx]

**Supplemental data**

**Supplemental figures**

**Supplemental Fig. 1: Homozygosity of Emiratis compared to world populations.** Plots similar to Figure 1 but using different maximum number of heterozygous calls allowed in the scanning windows. a, b; c, d; and e , f show plots with scanning windows having a maximum of 0; 1; 2 heterozygous call respectively. In plots g, h the window can contain up to 3 heterozygous calls for the low coverage sequencing data from the 1000 Genomes Project (The 1000 Genomes Project Consortium, et al. 2015) and a maximum of 1 heterozygous call for array data as suggested by Ceballos, Hazelhurst, et al. 2018.

**
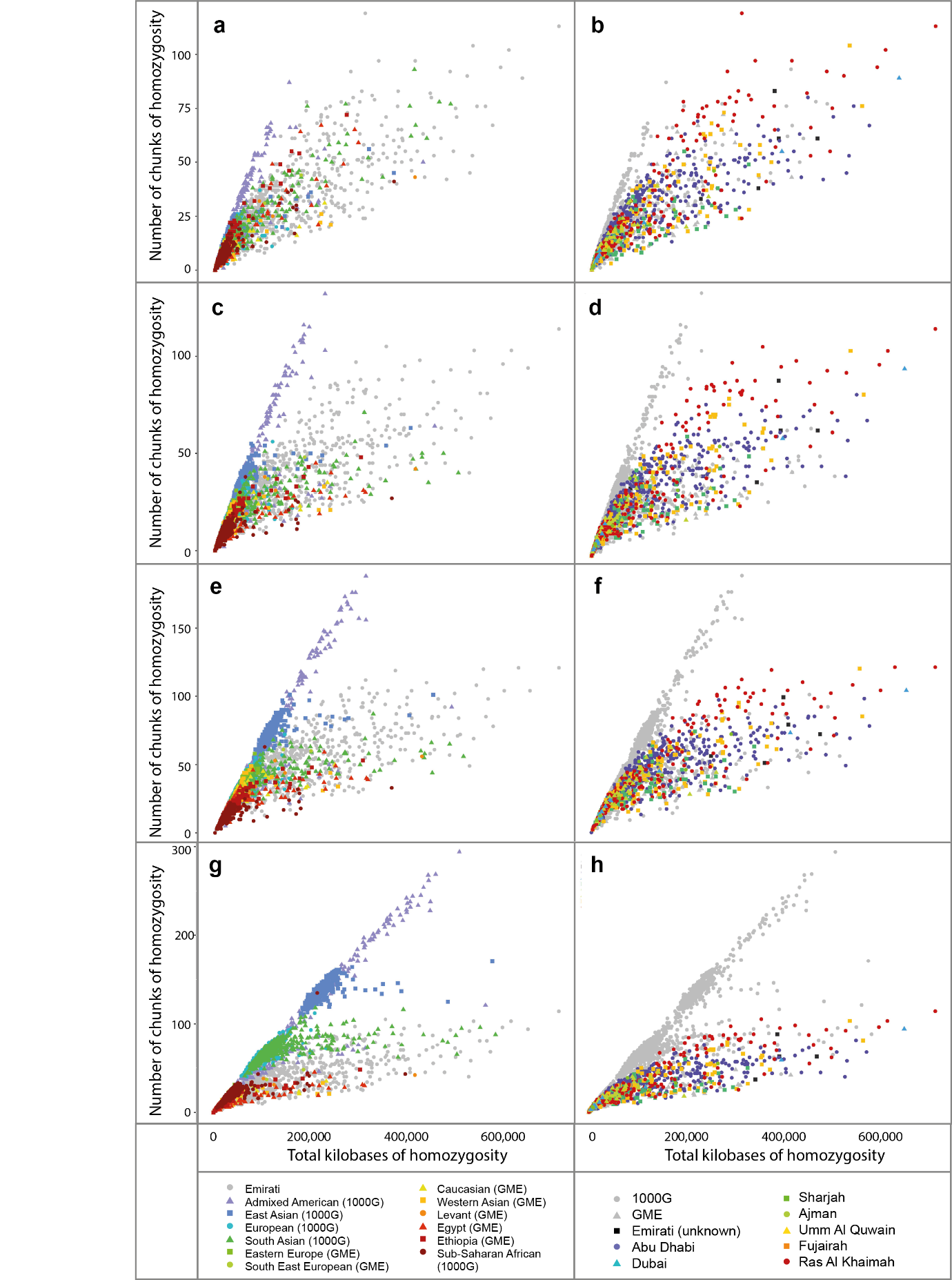
Supplemental Fig. 2: Kinship matrices. a.** Histograms (pihat) of 1000 Genomes superpopulations, GME populations and combined Emirati populations showing degree of relatedness between individuals. **b.** Equivalent histograms of individual Emirati populations. Abu Dhabi and Ras Al Khaimah Emirati populations show a degree of relatedness similar to that seen in Sub-Saharan Africans. All other Emirati populations show an even greater of relatedness. The plots illustrate the high degree of relatedness within Emirati populations reflecting their consanguineous and endogamous culture.


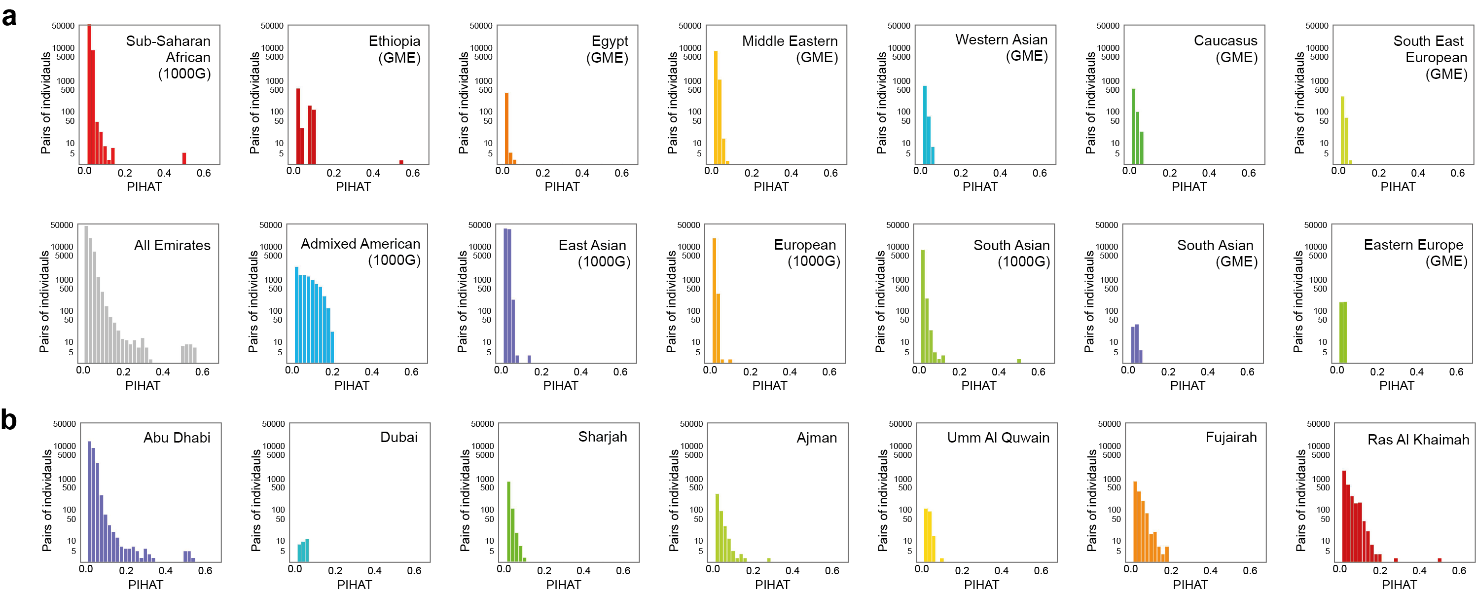


**Supplemental Fig 3. Intra and inter Emirati kinship.** Distribution of pairwise kinship (pihat) for the five largest Emirate sample groups. For each panel, the first box (white) shows intra-Emirate kinship matrices and the following coloured matrices show inter-Emirati matrices. This illustrates the greater degree of relatedness within individual Emirati populations compared to between Emirati populations consistent with endogamous culture. *p<0.05, **p<0.01, ***p<0.001, ****p<0.0001 (Bonferroni corrected)


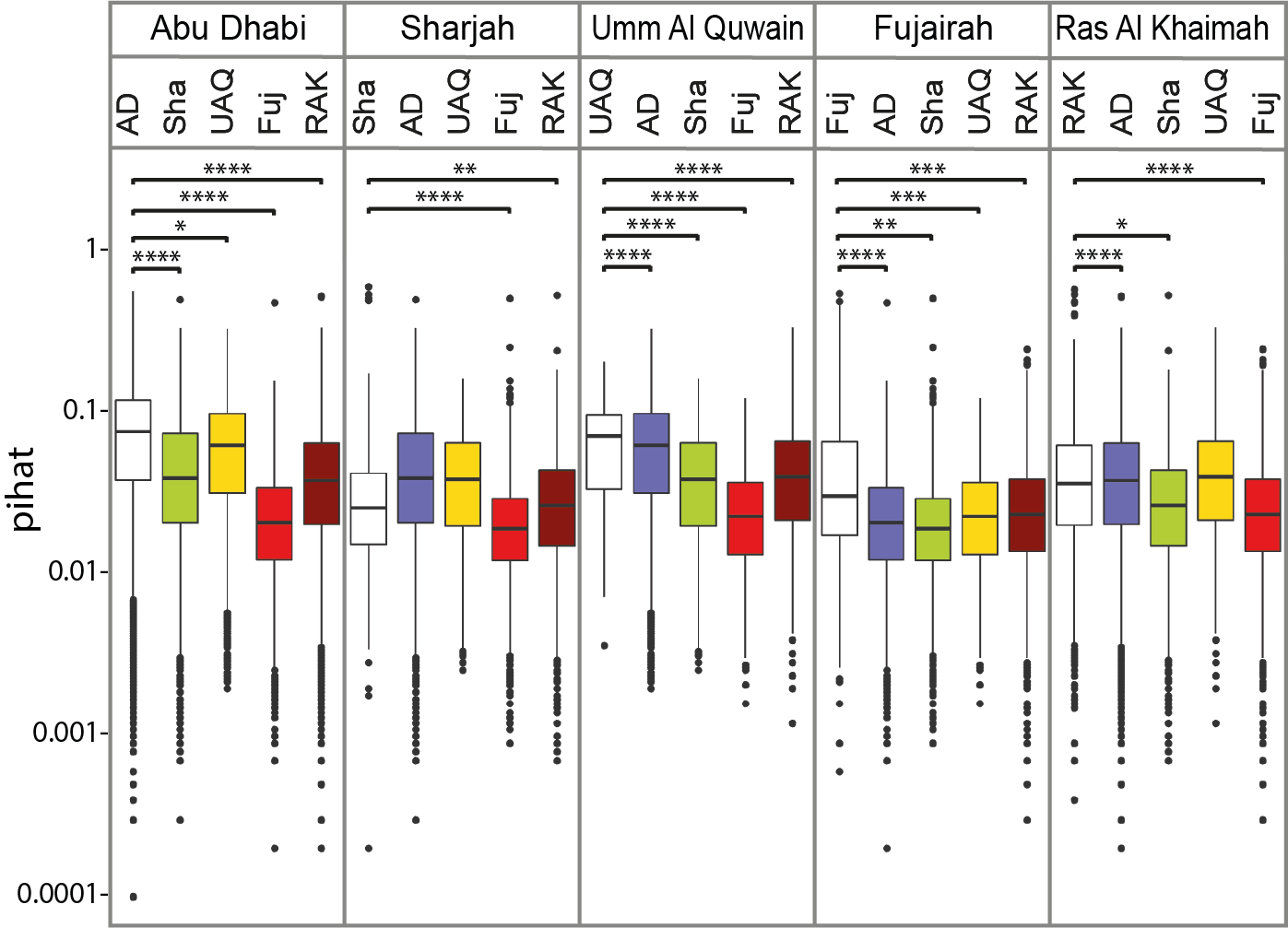


**Supplemental Fig. 4: Population tree.** Treemix analysis of the five largest Emirate samples with GME populations allowing five migration edges.

**
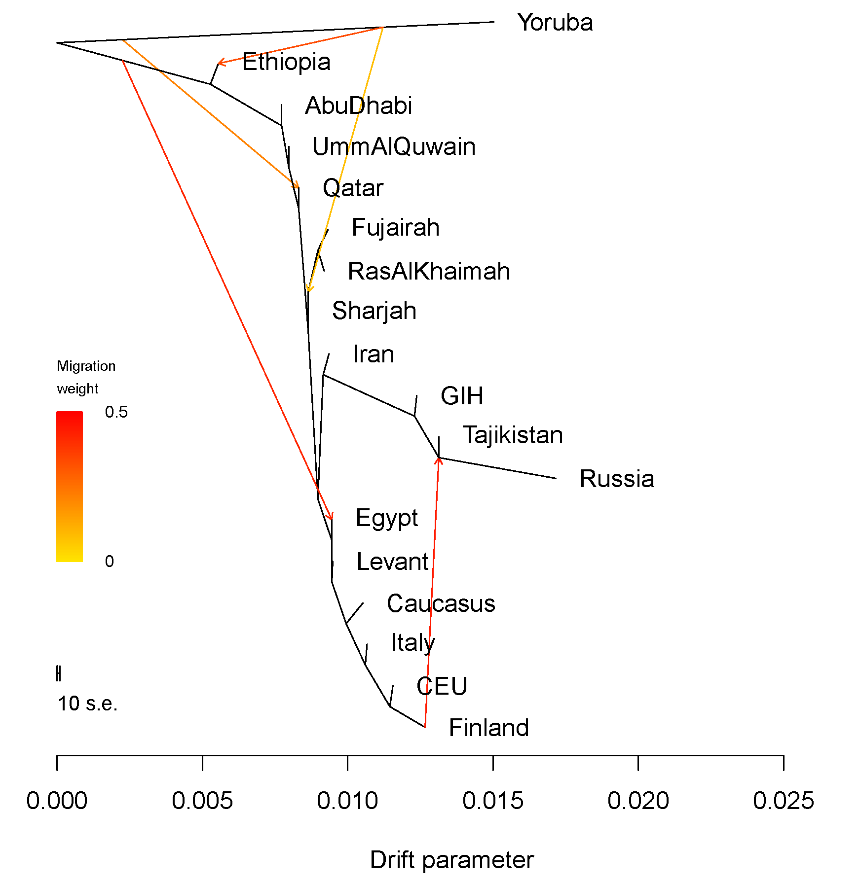
**

**Supplemental Fig. 5:** Time of admixture in the Emirates using ALDER. We use CEU and YRI as references to test admixture in a subset of Emiratis that represent the main population cluster (a) or a random subset from the Emirates (b). Similarly, we use CEU and ITU as references to test admixture in the main cluster (c) and a random subset (d). As expected, a random subset from the Emirates will always show more recent admixture from continuous gene flow into the population.


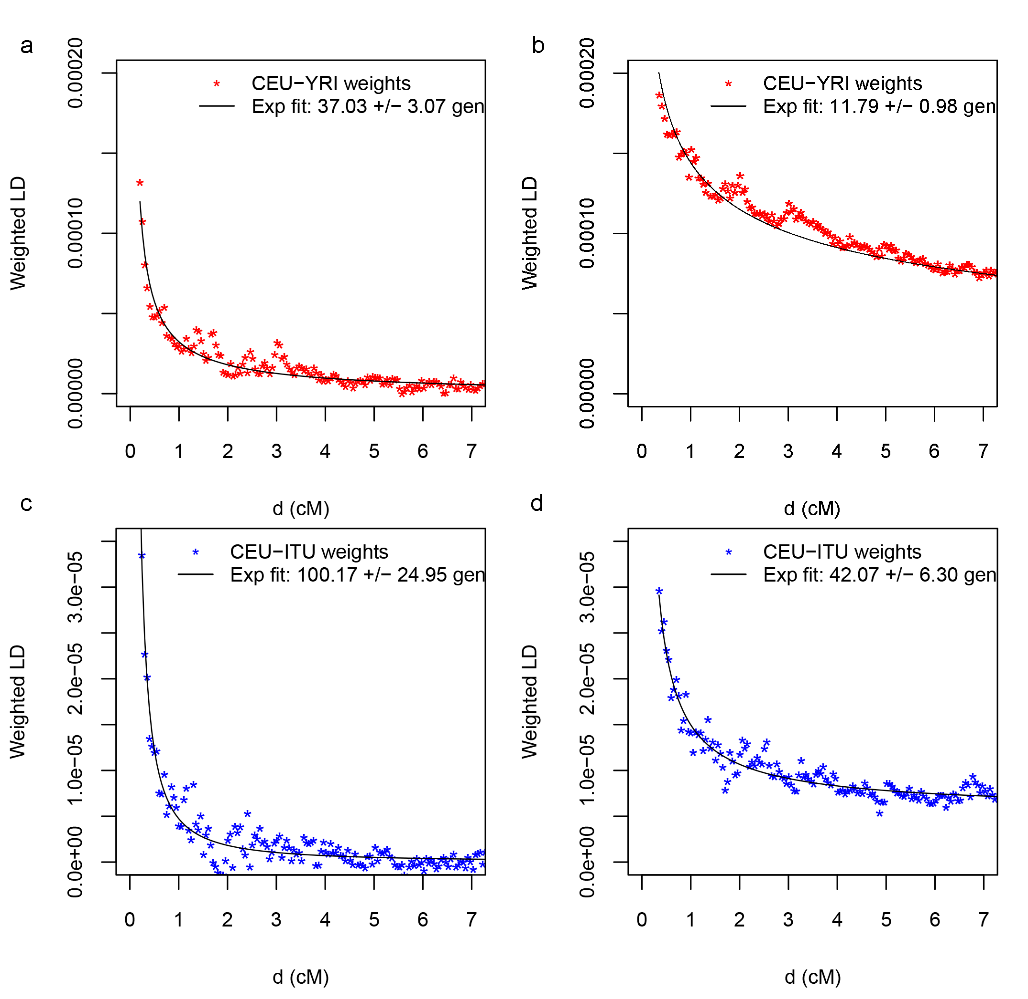


**Supplemental Fig. 6**: Shared ancestry between Emiratis and Greater Middle East and 1000G South Asian populations – Y chromosome and mitochondrial haplogroups. Heatmap of proportions of Y and mitochondrial haplogroups in each Emirate, tribe, GME population and 1000G superpopulation. The heatmap represents the proportion of each haplogroup in each group, for Y haplogroups in panel a and mitochondrial haplogroups in panel b. For example, Sharjah Emiratis show a greater heterogeneity in their Y haplogroups, compared to Fujairah Emiratis who have high prevalence of the R1b1a2a haplogroup. a. The majority of the Emirati Y haplogroups are dominated by the E1b1 haplogroup common to Africa and J1a2b-P58 prevalent in the Middle East and proximal regions. b. Mitochondrial haplogroups show a predominance of haplogroups U, K2, L, R0, H and HV. The contrast between the Emirates can also be seen in the mtDNA lineages. For example, haplogroup K2 is 28% in Ras Al Khaimah but just 4.3% in Abu Dhabi.

**
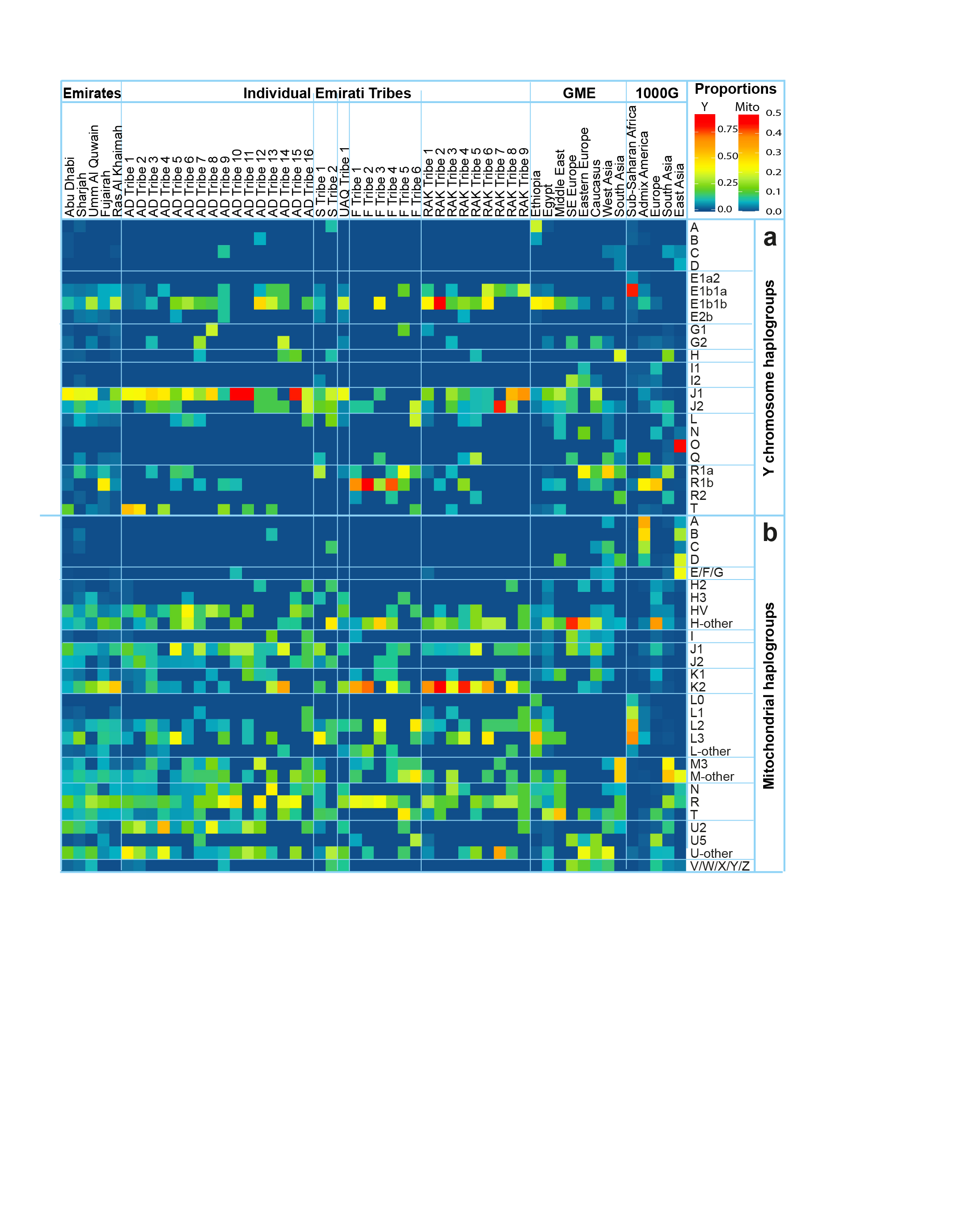
**

**Supplemental tables**

**Supplemental Table 1:** Per Emirati populations sample sizes and and sex. *Dubai and Ajman individuals were included in PCA and merged Emirati analyses, but excluded from other analyses due to small sample size and consequent lack of power.

| Emirate | Males | Females | Total |
| --- | --- | --- | --- |
| Abu Dhabi | 265 | 361 | 626 |
| Dubai* | 7 | 6 | 13 |
| Sharjah | 60 | 55 | 115 |
| Ajman* | 6 | 1 | 7 |
| Umm Al Quwain | 24 | 15 | 39 |
| Fujairah | 74 | 68 | 142 |
| Ras Al Khaimah | 130 | 115 | 245 |
| Unknown | 2 | 9 | 11 |
| Total | **568** | **630** | **1,198** |

**Supplemental Table 2: Datasets used for population analysis.** All datasets were used for PCA.

| Study | Data type | Number of variants | All individuals | Greater Middle East (GME) | Rest of the world |
| --- | --- | --- | --- | --- | --- |
| Emiratis * (this study) | SNP array | 1,058,982 | 1,198 | 1,198 | 0 |
| 1000 Genomes phase 3** (The 1000 Genomes Project Consortium, 2015) | Whole genomes | 68,382,305 | 2,504 | 0 | 2,504 |
| Ethiopians and Egyptians (Pagani, 2015) | Whole genomes | 32,652,305 | 220 | 100 | 120 |
| Jewish (Behar, 2010) | SNP array | 555,218 | 466 | 466 | 0 |
| Turkic speaking (Yunusbayev, 2015) | SNP array | 318,030 | 322 | 127 | 195 |
| Eurasians (Pagani, 2016) | Whole genomes | 37,508,402 | 402 | 96 | 306 |
| Total |  |  | **5,121** | **1,996** | **3,125** |

* see Table S2 for breakdown of Emiratis by Emirate ** see Table S3 for breakdown of 1000 Genomes phase 3 by 26 global populations

**Supplemental Table 3:** **1000 genomes phase 3 populations, super-populations and frequencies (The 1000 Genomes Project Consortium, 2015).**

| Population Code | Population Description | Super-population | # Samples |
| --- | --- | --- | --- |
| FIN | Finnish in Finland | Europe | 99 |
| CEU | Utah Residents (CEPH) with N & W Eur Ancestry | Europe | 99 |
| GBR | British in England and Scotland | Europe | 91 |
| TSI | Toscani in Italia | Europe | 107 |
| IBS | Iberian Population in Spain | Europe | 107 |
| GIH | Gujarati Indian from Houston, Texas | South Asia | 103 |
| PJL | Punjabi from Lahore, Pakistan | South Asia | 96 |
| BEB | Bengali from Bangladesh | South Asia | 86 |
| STU | Sri Lankan Tamil from the UK | South Asia | 102 |
| ITU | Indian Telugu from the UK | South Asia | 102 |
| CHB | Han Chinese in Beijing, China | East Asian | 103 |
| JPT | Japanese in Tokyo, Japan | East Asian | 104 |
| CHS | Southern Han Chinese | East Asian | 105 |
| CDX | Chinese Dai in Xishuangbanna, China | East Asian | 93 |
| KHV | Kinh in Ho Chi Minh City, Vietnam | East Asian | 99 |
| YRI | Yoruba in Ibadan, Nigeria | Africans | 108 |
| LWK | Luhya in Webuye, Kenya | Africans | 99 |
| GWD | Gambian in Western Divisions in the Gambia | Africans | 113 |
| MSL | Mende in Sierra Leone | Africans | 85 |
| ESN | Esan in Nigeria | Africans | 99 |
| ASW | Americans of African Ancestry in SW USA | Africans | 61 |
| ACB | African Caribbeans in Barbados | Africans | 96 |
| PUR | Puerto Ricans from Puerto Rico | Admixed American | 104 |
| MXL | Mexican Ancestry from Los Angeles USA | Admixed American | 64 |
| CLM | Colombians from Medellin, Colombia | Admixed American | 94 |
| PEL | Peruvians from Lima, Peru | Admixed American | 85 |

**Supplemental Table 4: Greater Middle East populations used for PCA (1000 genomes phase 3 populations shown in table S3).**

| Country | number of individuals | GME superpopulation | Number of inds in GME super-population |
| --- | --- | --- | --- |
| Azerbaijan(Pagani, et al. 2016) | 2 | Caucasus | 70 |
| Georgia(Pagani, et al. 2016) | 5 |  |  |
| Armenia(Pagani, et al. 2016) | 9 |  |  |
| Russia(Pagani, et al. 2016) | 26 |  |  |
| Russia(Yunusbayev, et al. 2015) | 28 |  |  |
| Hungary(Pagani, et al. 2016) | 1 | Eastern Europe | 31 |
| Latvia(Pagani, et al. 2016) | 3 |  |  |
| Lithuania(Pagani, et al. 2016) | 3 |  |  |
| Belarus(Pagani, et al. 2016) | 4 |  |  |
| Poland(Pagani, et al. 2016) | 4 |  |  |
| Estonia(Pagani, et al. 2016) | 6 |  |  |
| Ukraine(Pagani, et al. 2016) | 10 |  |  |
| Lebanon(Pagani, et al. 2016) | 1 | Middle East | 382 |
| Jordan(Pagani, et al. 2016) | 2 |  |  |
| Saudi Arabia(Pagani, et al. 2016) | 2 |  |  |
| Israel(Pagani, et al. 2016) | 8 |  |  |
| Levant(Behar, et al. 2010) | 369 |  |  |
| Egypt(Pagani, et al. 2012) | 100 | Egypt | 100 |
| Ethiopia(Pagani, et al. 2012) | 120 | Ethiopia | 120 |
| Moldova(Pagani, et al. 2016) | 2 | South Eastern Europe | 36 |
| Albania(Pagani, et al. 2016) | 3 |  |  |
| Bosnia-Herzegovina(Pagani, et al. 2016) | 7 |  |  |
| Cyprus(Behar, et al. 2010) | 12 |  |  |
| Moldova(Yunusbayev, et al. 2015) | 12 |  |  |
| Iran(Pagani, et al. 2016) | 4 | Central Asia | 116 |
| Kyrgyzstan(Pagani, et al. 2016) | 4 |  |  |
| Kazakhstan(Pagani, et al. 2016) | 6 |  |  |
| Uzbekistan(Pagani, et al. 2016) | 6 |  |  |
| Tajikistan(Pagani, et al. 2016) | 10 |  |  |
| Azerbaijan(Yunusbayev, et al. 2015) | 2 |  |  |
| Kazakhstan(Yunusbayev, et al. 2015) | 2 |  |  |
| Turkmenistan(Yunusbayev, et al. 2015) | 8 |  |  |
| Kyrgyzstan(Yunusbayev, et al. 2015) | 10 |  |  |
| Afghanistan(Yunusbayev, et al. 2015) | 11 |  |  |
| Uzbekistan(Yunusbayev, et al. 2015) | 12 |  |  |
| Iran(Yunusbayev, et al. 2015) | 16 |  |  |
| Tajikistan(Yunusbayev, et al. 2015) | 25 |  |  |

**Supplemental Table 5: Populations used for shared ancestry fineSTRUCTURE and ChromoPainter analysis.**

| Country/population | number of individuals | merged population | Number of individuals in merged population |
| --- | --- | --- | --- |
| Gambia (The 1000 Genomes Project Consortium, et al. 2015) | 113 | West Africa | 405 |
| Nigeria (Esan) (The 1000 Genomes Project Consortium, et al. 2015) | 99 |  |  |
| Nigeria (Yoruba) (The 1000 Genomes Project Consortium, et al. 2015) | 108 |  |  |
| Sierra Leone (Mende) (The 1000 Genomes Project Consortium, et al. 2015) | 85 |  |  |
| Kenya (Luhya) (The 1000 Genomes Project Consortium, et al. 2015) | 99 | Kenya | 99 |
| Ethiopian(Pagani, et al. 2012) | 120 | Ethiopia | 120 |
| Egypt(Pagani, et al. 2012) | 100 | Egypt | 100 |
| Israel(Pagani, et al. 2016) | 8 | Levant | 11 |
| Jordan(Pagani, et al. 2016) | 2 |  |  |
| Lebanon(Pagani, et al. 2016) | 1 |  |  |
| Italy (Toscani) (The 1000 Genomes Project Consortium, et al. 2015) | 107 | Southern Europe | 214 |
| Spain (Iberia) (The 1000 Genomes Project Consortium, et al. 2015) | 107 |  |  |
| Albania(Pagani, et al. 2016) | 3 | South Eastern Europe | 12 |
| Bosnia-Herzegovina(Pagani, et al. 2016) | 7 |  |  |
| Moldova(Pagani, et al. 2016) | 2 |  |  |
| Belarus(Pagani, et al. 2016) | 4 | Eastern Europe | 31 |
| Estonia(Pagani, et al. 2016) | 6 |  |  |
| Hungary(Pagani, et al. 2016) | 1 |  |  |
| Latvia(Pagani, et al. 2016) | 3 |  |  |
| Lithuania(Pagani, et al. 2016) | 3 |  |  |
| Poland(Pagani, et al. 2016) | 4 |  |  |
| Ukraine(Pagani, et al. 2016) | 10 |  |  |
| Armenia(Pagani, et al. 2016) | 9 | Caucasus | 42 |
| Azerbaijan(Pagani, et al. 2016) | 2 |  |  |
| Georgia(Pagani, et al. 2016) | 5 |  |  |
| Russia(Pagani, et al. 2016) | 26 |  |  |
| Iran(Pagani, et al. 2016) | 4 | Iran | 4 |
| Kazakhstan(Pagani, et al. 2016) | 6 | Central Asian GME | 26 |
| Kyrgyzstan(Pagani, et al. 2016) | 4 |  |  |
| Tajikistan(Pagani, et al. 2016) | 10 |  |  |
| Uzbekistan(Pagani, et al. 2016) | 6 |  |  |
| Bengal (Bangladesh) | 86 | South Asia | 514 |
| India(Pagani, et al. 2016) | 25 |  |  |
| India (Gujarati) (The 1000 Genomes Project Consortium, et al. 2015) | 103 |  |  |
| India (Telugu) (The 1000 Genomes Project Consortium, et al. 2015) | 102 |  |  |
| Pakistan (Pujabi) (The 1000 Genomes Project Consortium, et al. 2015) | 96 |  |  |
| sSri Lanka (Tamil) (The 1000 Genomes Project Consortium, et al. 2015) | 102 |  |  |

**Supplemental Table 6: Emirate Y and mitochondrial haplogroups.** Excel spreadsheet (Y_chr_and_mito_genotypes_Illumina_Multi_Ethnic_Global_Array_variants.xlsx) shows Y chromosome and mitochondrial haplogroups for each Emirati with abbreviated groups used for heat map plot. Also shown are the haplogroup mutations on the Illumina Multi Ethnic Global Array used for genotyping. Alleles shown as “0” correspond to alleles not found in our dataset.

Behar DM, Yunusbayev B, Metspalu M, Metspalu E, Rosset S, Parik J, Rootsi S, Chaubey G, Kutuev I, Yudkovsky G, et al. 2010. The genome-wide structure of the Jewish people. Nature 466:238-242.

Pagani L, Kivisild T, Tarekegn A, Ekong R, Plaster C, Gallego Romero I, Ayub Q, Mehdi SQ, Thomas MG, Luiselli D, et al. 2012. Ethiopian genetic diversity reveals linguistic stratification and complex influences on the Ethiopian gene pool. Am J Hum Genet 91:83-96.

Pagani L, Lawson DJ, Jagoda E, Morseburg A, Eriksson A, Mitt M, Clemente F, Hudjashov G, DeGiorgio M, Saag L, et al. 2016. Genomic analyses inform on migration events during the peopling of Eurasia. Nature 538:238-242.

The 1000 Genomes Project Consortium, Auton A, Brooks LD, Durbin RM, Garrison EP, Kang HM, Korbel JO, Marchini JL, McCarthy S, McVean GA, et al. 2015. A global reference for human genetic variation. Nature 526:68-74.

Yunusbayev B, Metspalu M, Metspalu E, Valeev A, Litvinov S, Valiev R, Akhmetova V, Balanovska E, Balanovsky O, Turdikulova S, et al. 2015. The genetic legacy of the expansion of Turkic-speaking nomads across Eurasia. PLoS Genet 11:e1005068.
